# Patients’ satisfaction with long-acting injectable somatostatin analog therapy for neuroendocrine tumors

**DOI:** 10.1186/s41687-021-00355-5

**Published:** 2021-09-07

**Authors:** Christina Darden, Mark Price, David Ray, Grace Goldstein, Diana Goss, Lee Bennett, Diana Garbinsky, Ramya Thota

**Affiliations:** 1 Department of Surveys and Observational Studies, RTI Health Solutions, Research Triangle Park, NC USA; 2 Ipsen Biopharmaceuticals, Inc., Cambridge, MA USA; 3 Carcinoid Cancer Foundation, Mount Kisco, NY USA; 4 Department of Patient-Reported Outcomes, RTI Health Solutions, Research Triangle Park, NC USA; 5 Department of Biometrics, RTI Health Solutions, Research Triangle Park, NC USA; 6 Intermountain Medical Center, Murry, UT USA

**Keywords:** Neuroendocrine tumor, Serial survey, Patient experience, Satisfaction, Interviews, Somatostatin analog

## Abstract

**Background:**

Long-acting somatostatin analogs (LA SSAs) are approved and recommended for the treatment of patients with advanced neuroendocrine tumors (NETs). Given the long duration of therapy and differences in administration routes, it is important to understand patients’ experiences with receiving LA SSA injections.

**Methods:**

We conducted a serial survey, informed by qualitative interviews with eight patients treated with LA SSAs and two nurses who administer LA SSA injections, among patients undergoing LA SSA treatment over a 28-day period (administered at baseline and 14 days and 28 days after injection). Eligible patients, recruited by the Carcinoid Cancer Foundation, self-reported having received an LA SSA injection for physician-diagnosed NET within the 5 days before the survey.

**Results:**

202 patients completed the survey at baseline (82 receiving lanreotide and 120 receiving octreotide), 148 at day 14, and 124 at day 28. Patients reported consistently high satisfaction levels with their most recent LA SSA injection (91.1% at baseline, 85.1% at day 14, and 85.5% at day 28); 68.8% reported that their injection experience differed based on the nursing staff administering the injection.

**Conclusions:**

Satisfaction with LA SSA injections is high among patients in this population, and specific experiences with LA SSA injections varied based on the nursing staff administering the injection. Evaluations of patients’ experiences and satisfaction with treatment are increasingly important as patients take more active roles in decision-making for their treatment pathways.

## Introduction

Although neuroendocrine tumors (NETs) can occur anywhere in the body, approximately two-thirds occur in the gastrointestinal system, most commonly in the small intestine or appendix. The incidence of NETs is low, albeit increasing: the annual age-adjusted incidence of NETs in the United States (US) was estimated to be 1.09 per 100,000 persons in 1973 and 6.98 per 100,000 persons in 2012 [1]. However, the prevalence of NETs is relatively high and is also increasing; the estimated 20-year limited-duration prevalence in 1993 was 0.006% and in 2012 was 0.048%. Neuroendocrine tumors are generally slow-growing and often diagnosed at an advanced stage of disease [2]. While often asymptomatic, NETs may be associated with a paraneoplastic condition called carcinoid syndrome (CS) that can develop in patients who have functional NETs [3, 4]. The most common manifestations of CS include diarrhea, skin flushing, facial skin lesions, wheezing or difficulty breathing, rapid heartbeat, and right-sided valvular heart disease or heart failure [4, 5].

Owing to the indolent and often unresectable nature of NETs, many patients undergo long-term systemic therapy [6]. Approved or recommended treatments for NETs and/or the associated symptoms include the long-acting (LA) somatostatin analogs (SSAs) octreotide (administered by intramuscular injection every 4 weeks) or lanreotide (administered by deep subcutaneous injection every 4 weeks), targeted therapy such as sunitinib, everolimus, peptide receptor radioactive therapy, and chemotherapy such as capecitabine, temozolomide [7, 8]. SSAs are the mainstay of treatment for functional NETs in the management of CS [7, 8]. In addition to treatment of the underlying cancer, patients with CS typically receive SSAs to control associated symptoms (e.g., diarrhea and flushing) [5]; SSAs are also used for tumor control in advanced, nonfunctional, well-differentiated NETs. While duration of SSA therapy varies based on patient and tumor characteristics, many patients may undergo treatment for 5 years or longer [9]. While evidence on the long-term impact of SSA therapy on patients’ quality of life (QOL) is somewhat limited, a study evaluating QOL among 87 patients with CS using the Functional Assessment of Cancer Therapy-General (FACT-G) observed clinically meaningful QOL improvements among patients who had received SSA therapy for 2 years or less but not among patients receiving therapy for a longer duration [9].

Given the long duration of SSA therapy for patients with NET, it is important to understand patients’ injection experiences and the factors that affect their satisfaction with treatment; however, limited evidence exists on patients’ experiences and satisfaction with SSA therapy. The objective of this study was to describe patients’ real-world experiences with the modes of administration for the LA formulations of octreotide and lanreotide and to evaluate how patient experiences may affect satisfaction with these treatments over time.

## Methods

This was a prospective, serial, web-based survey study conducted using both qualitative and quantitative methods among patients with a self-reported physician diagnosis of a NET in the US. Overall, the study aimed to assess patient satisfaction with LA SSA treatment (octreotide or lanreotide) over 28 days. Phase 1 of the study, survey instrument development, was predominantly qualitative and informed by interviews with patients and nurses to solicit feedback on the clarity and comprehensiveness of the survey. Phase 2 of the study focused on quantifying the results of the survey. Development of the survey instrument and analysis of the survey data followed standard survey methodological principles and best practices, described in further detail in “Phase 1: survey instrument development” and “Phase 2: survey administration, analysis, and reporting” sections. While CS is a sequela of a rare disease, it is important to follow standard processes for survey development to ensure that the resulting instrument is easily understood and functions as expected with the intended survey population.

The study was reviewed and approved by the RTI International institutional review board. All study participants provided electronic informed consent.

### Phase 1: survey instrument development

The survey instrument was designed by researchers, medical experts, and a representative from the Carcinoid Cancer Foundation (CCF) to capture patient satisfaction with LA SSA injections, focusing on differences in route of administration. While formal validation of the survey instrument for future use was not intended, development of the survey instrument followed rigorous qualitative and quantitative practices.

Standard survey methodological principles were used to draft an initial set of survey items. Qualitative interviews then were conducted with convenience samples of patients and nurses, recruited by CCF, to cognitively pretest a draft version of the survey instrument, refine the survey items and response options, and identify and/or expand on additional concepts potentially relevant for the measurement of patient satisfaction with LA SSA injections. Cognitive interviews are a well-established qualitative research methodology used to identify problems with and refine the questionnaire items and response options [10]. Using a semistructured guide, 1-h concept elicitation and cognitive pretesting interviews were conducted with eight patients with NET and experience receiving LA SSAs and two nurses with LA SSA injection experience. Standard “think aloud” procedures were used with directed probes to delve further into the question/answer process and to explore iterative changes. Participants interpreted the items (paraphrasing key sentences in the participants’ own words) and what they thought about suggested responses and scales. Participants also described personal strategies for preparing for injections and minimizing or preventing any after affects. After completion of the interviews, the survey instrument was revised and finalized.

### Phase 2: survey administration, analysis, and reporting

The final survey instrument measured patient satisfaction and experience with LA SSA injections over a 28-day period, in addition to collecting data on patient demographics, treatment, and medical history. Respondents completed surveys on prior/current experience and satisfaction at baseline, 14 days after injection, and 28 days after injection but before next injection. Patients who completed the baseline survey and did not report having received another LA SSA injection after completion of the baseline survey were eligible to complete both the day 14 and the day 28 surveys. For the day 14 and day 28 surveys, e-mail reminders were sent to potential respondents each day following the invitation for 3 days. Respondents had a 5-day window to complete the baseline questionnaire and 4-day windows to complete the day 14 and day 28 surveys.

The number of questions in the baseline, day 14, and day 28 survey instruments varied based on skip logic. Excluding screening, consent, and e-mail change questions, the baseline survey contained a maximum of 61 questions and the day 14 and day 28 surveys contained a maximum of 19 questions. The baseline survey took approximately 20 min to complete, and the day 14 and day 28 surveys took approximately 5 min to complete. The Qualtrics survey management system was used to collect and host survey data. Data were extracted from Qualtrics to SPSS and then converted to SAS after dropping unnecessary variables.

#### Study population

Patients eligible to complete the survey were aged ≥ 18 years with a self-reported physician diagnosis of NET, with or without CS diagnosis or symptoms (including diarrhea, skin flushing, abdominal pain/cramps, wheezing, shortness of breath or breathing difficulties, rapid heartbeat, and night sweats), who self-reported having received their dose of either LA octreotide or lanreotide as a single injection (vs. multiple injections) within the 5 days before taking the survey for the treatment of their NET or to treat or prevent symptoms of CS. For comparison purposes, patients who self-reported multiple injections were excluded.

Patients were identified and recruited by CCF. Members of CCF’s e-newsletter group were invited to participate in the study via e-mail. Survey screening questions were used to assess potential respondents’ eligibility. Assuming a sample size of 200 subjects, a two-sided 95% confidence interval for a sample proportion using the normal approximation would extend 7% from the observed proportion for an expected proportion of 50%; thus, the study targeted 200 completed baseline surveys.

#### Statistical analyses

All analyses were descriptive in nature. Results were presented by study time point (baseline, day 14 and day 28), where applicable. Any respondent with missing data for the day 14 or day 28 questionnaire was excluded from any analysis for that time point. All point estimates were accompanied by the appropriate measure of variance. Responses of “Prefer not to answer” and “Not applicable” were excluded from the denominator for computation of each proportion, and missing data were not imputed. All analyses were performed in SAS version 9.4 (SAS Institute, Cary, North Carolina).

## Results

### Phase 1: survey instrument development

Results from the qualitative cognitive debriefing interviews with patients and nurses that informed development of the survey revealed several concepts related to the importance of LA SSA injection to patients with NET, including nurse training and familiarity with the injection process and proper preparation and administration of the injection. Among the eight patients participating in the qualitative interviews, five were receiving lanreotide treatment and three were receiving octreotide treatment at the time of the interview.

Patients reported variable experiences across their prior SSA injections. Most patients expressed confidence in their “usual” or familiar injection nurse, reporting minimal, familiar, or expected levels of pain or discomfort. Most also were skeptical of unfamiliar nurses and their knowledge about injection preparation and administration. Patients were apprehensive particularly when they observed unfamiliar or inconsistent preparation practices (e.g., a different mixing or preparation, different body position for the injection, different skin pinching or flattening). Several patients described preinjection and/or postinjection actions taken to alleviate physical discomfort (e.g., soreness, pain) associated with injections. These included topical anesthetic on injection site before injection; allowing an alcohol wipe to dry before the injection so that alcohol is not pushed into the skin, causing pain; over-the-counter pain reliever (e.g., acetaminophen or naproxen); ice cubes/hot pad on injection site; acupuncture; cannabis; and lying posture on the table with toes inward to relax hip/buttocks.

The two nurses participating in the qualitative interviews described various factors affecting their injection approaches. Patient and clinic decision-making factors in prescribing LA octreotide versus LA lanreotide included patient body mass index and patient experience with side effects; specifically, those with more diarrhea may be prescribed LA octreotide. Nurses also noted that the need to bring both octreotide and lanreotide injections to room temperature affects the injection preparation time. Some nurses described setting out medications on the counter before patient arrival to shorten the patient wait time. Nurses also described the need to time the octreotide saturation correctly before the liquid crystallizes and clogs the needle or substitute the octreotide needle from the kit for the original gauge needle to avoid the clogging. One nurse commented that “Somatuline does not require much prep time.”

Results of the cognitive debriefing interviews found that the survey was well understood and captured relevant concepts related to SSA injection. Minor revisions were made to the terminology of the survey questions to promote clarity and understanding among survey respondents; for example, both the generic and the brand names of the treatments of interest were included in the questionnaire, and a question asking about respondents’ experience with CS was refined to include not only a diagnosis of CS but also its hallmark symptoms.

### Phase 2: survey administration, analysis, and reporting

#### Respondent characteristics

Study invitations and reminders were first sent via e-mail to 2122 members of the CCF e-newsletter group identified as being associated with NET patients. A second set of e-mail invitations and reminders was sent to a random sample of CCF’s e-newsletter membership list (5000 e-mail addresses). A total of 200 baseline surveys were targeted and 202 were completed; the survey was then closed after the completion target was reached, for a response rate of 3%.

**Fig. 1:**
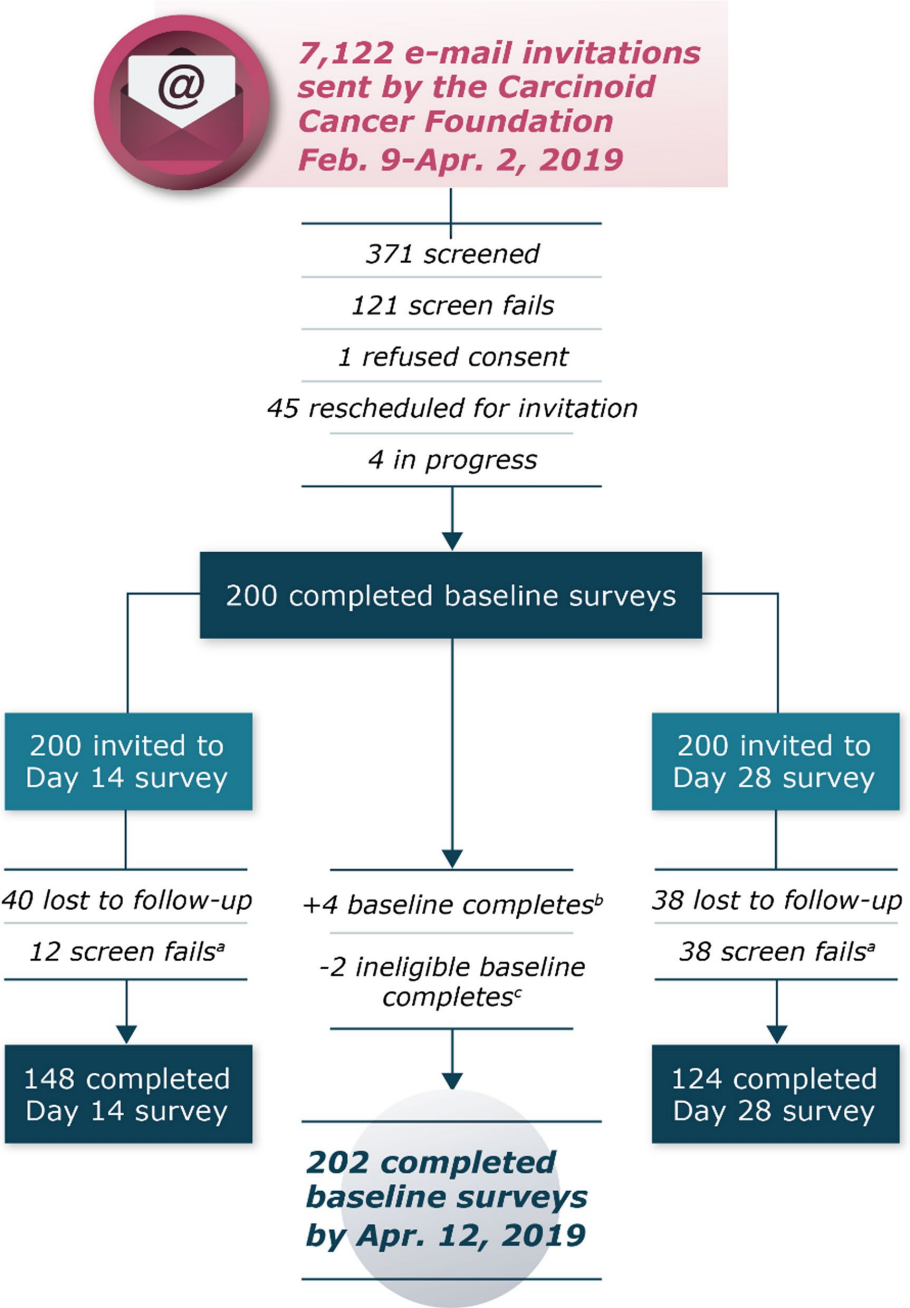
Survey disposition. LA SSA = long-acting somatostatin analog. ^a^Reported receipt of another LA SSA since baseline. ^b^Four completed but not submitted baseline surveys were moved to completed status at the end of data collection. ^c^There were 2 patients who screened into the baseline survey, which indicated that it had been less than 5 days since their most recent LA SSA injection. These patients later provided the date of their last injection as occurring 15 and 25 days before taking the survey. The completed surveys for these 2 patients were not included in the analysis data set

Of the 202 patients who completed the baseline survey (82 receiving lanreotide and 120 receiving octreotide), 148 completed the survey on day 14 (56 lanreotide, 92 octreotide), and 124 completed the survey on day 28 (51 lanreotide, 73 octreotide) (Fig. 1). Patients had a mean (standard deviation [SD]) age of 63.2 (9.9) years; a majority were female (61.9%) and white (93.1%), and 48.0% were retired (Table 1). More than half of the patients (53.0%) had a university or graduate degree. A total of 84 patients (41.6%) reported that a caregiver helped to manage their disease or treatment. Mean (SD) body mass index was 27.8 (6.1) kg/m^2^ for the total population. Most patients (57.4%) reported being fully active and able to perform all normal activities without restriction before their most recent LA SSA injection.

**Table 1:** Baseline patient characteristics (N = 202)

Characteristic	Patients (N = 202)
Age, mean (SD) years	63.2 (9.9)
Sex, female, n (%)	125 (61.9)
Body mass index,^a^ mean (SD)	27.8 (6.1)
Race or ethnicity, n (%)	
White or Caucasian	188 (93.1)
Black or African American	4 (2.0)
Hispanic or Latino	3 (1.5)
Other	2 (1.0)
American Indian or Alaska Native	1 (0.5)
> 1 race selected	3 (1.5)
Prefer not to answer	1 (0.5)
Region, n (%)	
South	65 (32.2)
Midwest	55 (27.2)
Northeast	41 (20.3)
West	41 (20.3)
Spouse/family member/caregiver/other adult currently help to manage disease or treatment, n (%)	84 (41.6)

*SD* Standard deviation

^a^N = 200

Table 2 presents patients’ treatment and medical history at baseline. The mean (SD) time since NET diagnosis was 6.9 (5.0) years. Most patients reported a gastrointestinal NET (n = 137, 67.8%), and 89.6% (n = 181) reported a diagnosis of CS or had experienced CS-related symptoms. Most patients reported receiving their most recent LA SSA injection at a community (nonacademic) center (50.0%) or academic or university-associated center (42.1%). The remainder reported that they received their injection at home by a visiting nurse (2.5%) or in another location (5.0%) or were unsure of the location of their most recent injection (0.5%). Approximately 50% of patients had been receiving their injection for 1 to < 5 years.

**Table 2:** Treatment and medical history

Characteristic	Patients (N = 202)
Time since NET diagnosis,^a^ mean (SD) years	6.9 (5.0)
Diagnosed with CS or experienced CS-related symptoms, n (%)	181 (89.6)
Type of LA SSA injection currently receiving, n (%)	
Somatuline depot (lanreotide)	82 (40.6)
Sandostatin LAR (octreotide)	120 (59.4)
Length of time receiving current injection, n (%)	
Less than a year	44 (21.8)
1 to < 5 years	98 (48.5)
5 to < 8 years	32 (15.8)
8 years or more	28 (13.9)
Experience receiving lanreotide and octreotide, n (%)^b^	41 (20.3)
Location of most recent injection, n (%)	
Community (nonacademic) clinic/office/treatment center	101 (50.0)
Academic or university-associated clinic/office/treatment center	85 (42.1)
At home by a visiting nurse	5 (2.5)
Other	10 (5.0)
Don’t know/not sure	1 (0.5)
Primary location of NET, n (%)	
Gastrointestinal^c^	137 (67.8)
Unknown primary site^d^	21 (10.4)
Lung	17 (8.4)
Pancreas	12 (5.9)
Liver^e^	3 (1.5)
Kidney	2 (1.0)
Ovary	1 (0.5)
Other	7 (3.5)
Don’t know/don’t remember	2 (1.0)

*CS* Carcinoid syndrome, *LA SSA* long-acting somatostatin analog, *LAR* long-acting release, *NET* neuroendocrine tumors; *SD* standard deviation

^a^N = 200

^b^Respondents were asked whether they had ever received octreotide and ever received lanreotide; 41 respondents had received both types of LA SSA

^c^Includes appendix (n = 8), large intestine (colon, large bowel) (n = 13), small intestine (small bowel, duodenum, jejunum, ileum) (n = 109), stomach (n = 5), and rectum (n = 2)

^d^The primary tumor site was unknown to the physician at diagnosis

^e^Primary NETs of the liver are extremely rare, and respondents with metastatic disease to the liver may have reported their tumors as being primary to the liver. Metastatic disease to the liver cannot be ruled out for these responses

#### Satisfaction with treatment

**Fig. 2:**
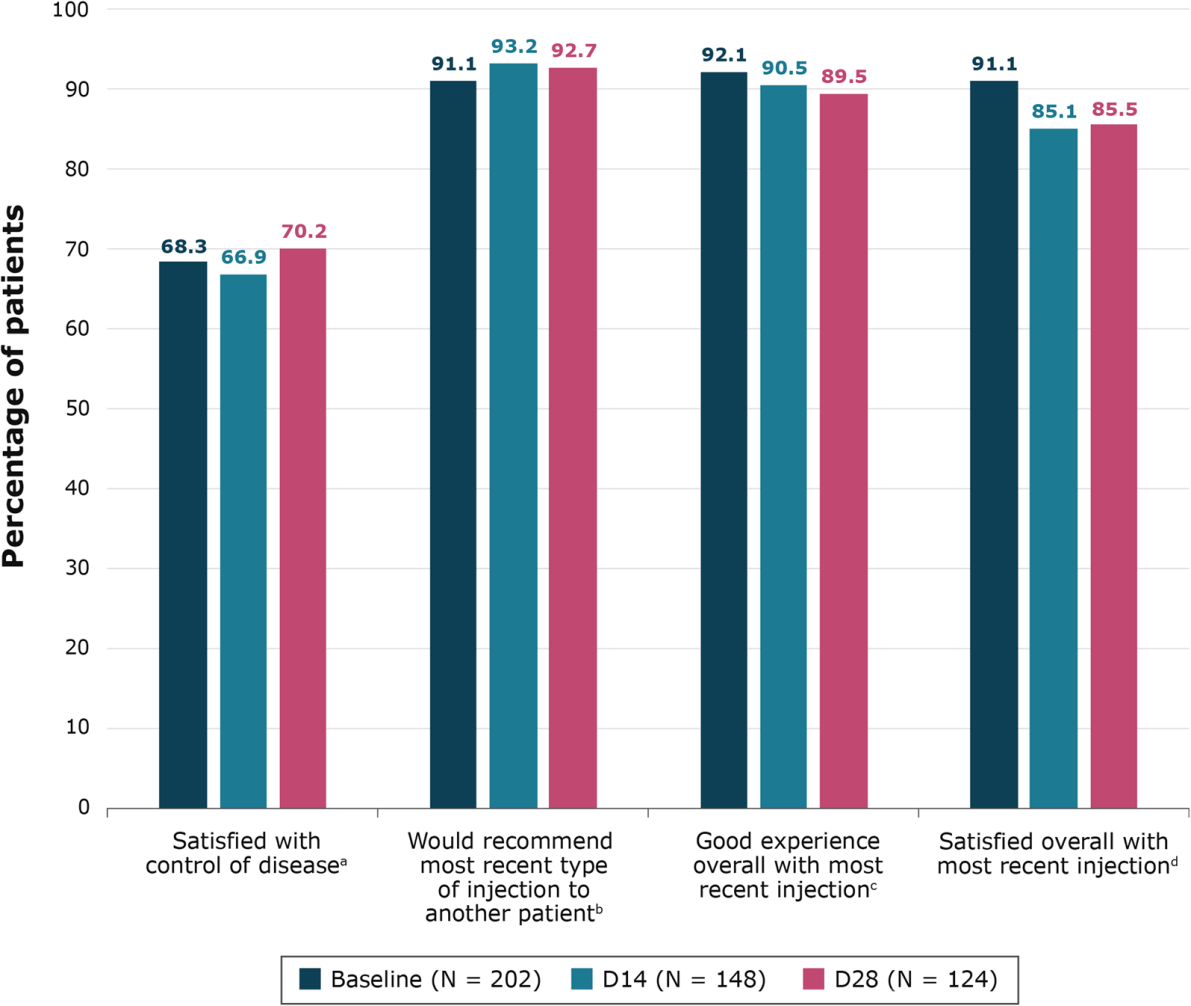
Overall patient satisfaction with treatment at baseline, day 14, and day 28. ^a^ Includes responses of “Very satisfied” or “Somewhat satisfied”. ^b^Includes response of “Definitely recommend” or “Probably recommend”. ^c^Includes responses of “Very good experience” or “Good experience”. ^d^Includes responses of “Very satisfied” or “Satisfied.”

Patients reported consistently high satisfaction levels (“very satisfied” or “somewhat satisfied” response options) with their most recent LA SSA injection (Fig. 2). Overall, 91.1% of patients (n = 184) were satisfied with their most recent injection at baseline (within 5 days following their most recent injection) (95% confidence interval [CI], 87.2–95.0%), 85.1% (n = 126) at day 14 (95% CI, 79.4–90.9%), and 85.5% (n = 106) at day 28 (95% CI, 79.3–91.7%). In addition, most patients reported that they were satisfied with how their injection was controlling their disease at baseline (68.3%, n = 138) (95% CI, 61.9–74.7%), day 14 (66.9%, n = 99) (95% CI, 59.3–74.5%), and day 28 (70.2%, n = 87) (95% CI, 62.1–78.2%). When asked if they would recommend their injection to another patient like themselves, 91.1% (n = 184) of patients at baseline reported that they would do so (95% CI, 87.2–95.0%), 93.2% (n = 138) at day 14 (95% CI, 89.2–97.3%), and 92.7% (n = 114/123) at day 28 (95% CI, 88.1–97.3%). Most patients reported a “good experience” or “very good experience” with their most recent injection at baseline (92.1%, n = 186) (95% CI, 88.4–95.8%), at day 14 (90.5%, n = 134) (95% CI, 85.8–95.3%), and at day 28 (89.5%, n = 111) (95% CI, 84.1–94.9%).

#### Treatment and injection experience

**Fig. 3:**
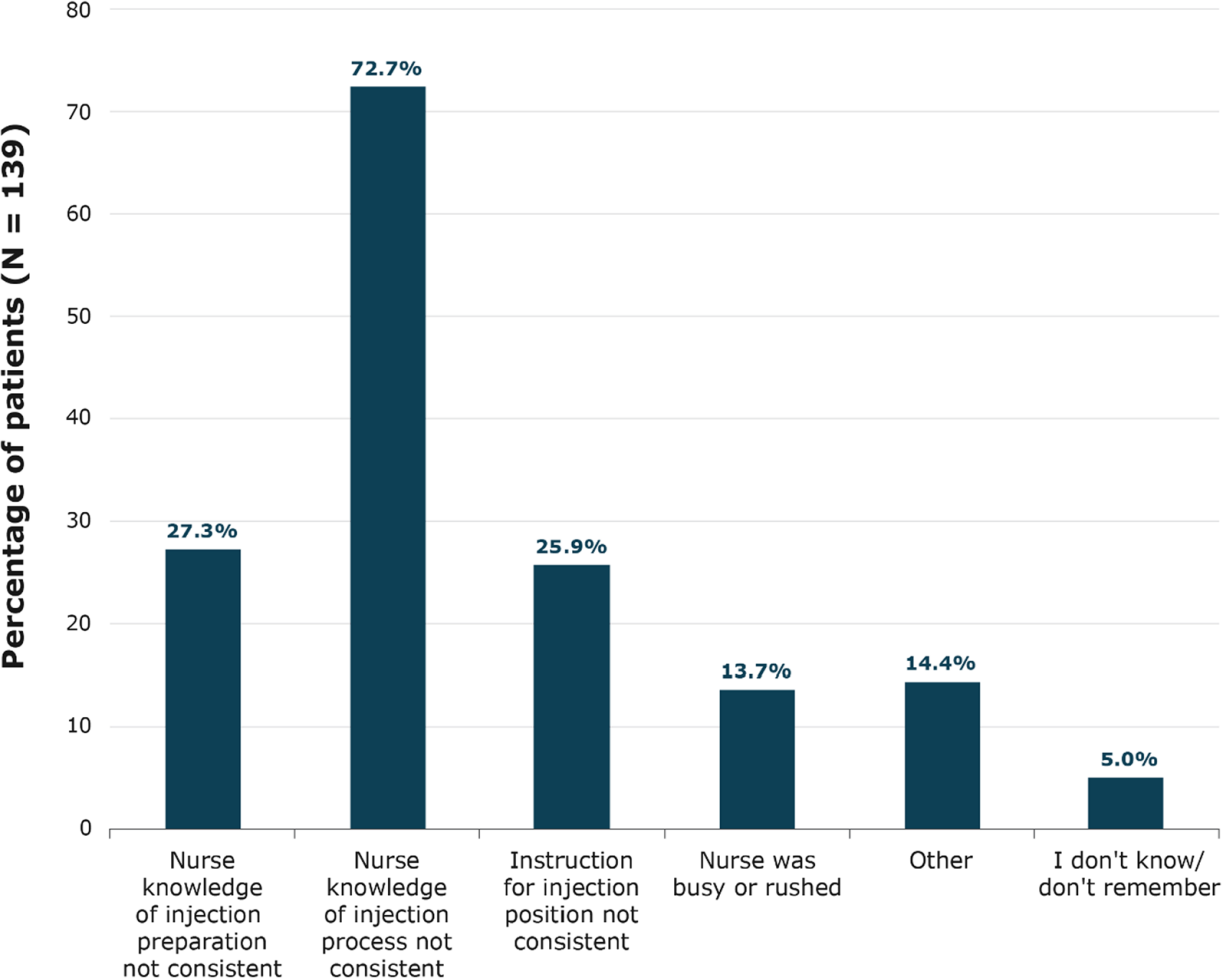
Patient-Reported Reasons Injection Process Differs by Nursing Staff

At baseline, patients were asked about their experiences with LA SSA injections. When asked how long they had to wait for a nurse to prepare their most recent injection, most patients (81.6%, n = 164) reported waiting < 30 min. At baseline, patients were asked if they took any actions before or after their injection. Most patients (80.2%; n = 162) reported that they took no actions, 9.4% (n = 19) reported taking an over-the-counter pain reliever, 5.0% (n = 10) used a hot pad, 4.5% (n = 9) used a topical anesthetic before or after their injection, 1.5% (n = 3) used an ice pack, and 3.5% (n = 7) reported “other” (multiple responses were permitted). At baseline, 68.8% (n = 139) of patients (95% CI, 62.4–75.2%) said that their monthly injections differed based on the nursing staff/person administering the injection. Of those patients, 72.7% (n = 101) (95% CI, 65.3–80.1%) said this was due to varying levels of nurse knowledge of injection processes (Fig. 3).

At baseline, most patients reported little (33.7%; n = 68) or no (57.4%; n = 116) anxiety immediately prior to their last injection. Among the 86 patients (42.6%) who reported experiencing any amount of anxiety, the most commonly reported reasons were “I experienced pain, swelling, bruising, soreness and/or a lump or knot at the injection site in the past” (46.5%; n = 40), “a previous injection did not go smoothly or as I expected” (33.7%; n = 29), and “I was not familiar with the nurse” (30.2%; n = 26).

Patients were also asked to think about the severity of the pain or discomfort at the injection site during their worst injection experience, as well as the severity of the pain or discomfort at the injection site during their most recent injection. Patients’ “worst injection experience” feedback was collected, regardless of time since that experience, as patients who participated in the cognitive interviews expressed how memorable poor injections are and future experiences are often measured against their worst reference point. Referencing their worst experience, only a small percentage reported mild (23.8%; n = 48 of 202) to no pain or discomfort (6.4%; n = 13 of 202) at the injection site during the injection. At the time of the survey, 64.4% (n = 130 of 202) of patients reported mild to no pain or discomfort at the injection site during the injection at baseline (within 5 days of their most recent injection). After 14 days, 70.3% (n = 104 of 148) recalled mild to no pain or discomfort at the injection site during the injection, and after 28 days, 70.2% (n = 87 of 124) recalled mild to no pain or discomfort at the injection site during the injection.

## Discussion

In general, patients with NET were satisfied overall with their most recent injection of LA octreotide or LA lanreotide (91.1% at baseline, 85.1% at day 14, and 85.5% at day 28). Patients reported a good overall experience with the most recent injection, which was consistently high over the 28 days (89.5–92.1%), and they reported that they would recommend their type of injection to another patient like themselves. However, patient satisfaction with how their injection was controlling their disease was slightly lower (66.9–70.2%) but remained consistent across the month.

As noted in a prior study [11], as an emergent theme in the NET patient interviews [12], and as observed in this study’s results, patient injection experiences vary greatly from injection to injection and are influenced by the nurse(s) administering the injections. A majority of patients, 69%, indicated that their monthly injections differed based on the nursing staff/person administering the injection. These patients felt that this was because of varying levels of nurse knowledge of the injection process (73% of patients), knowledge of injection preparation (27%), or because the nurse’s instruction for injection body position was not consistent (26%). These inconsistencies, as noted by patients, are likely contributing factors to patients’ differing experiences. Further, several patients who participated in qualitative interviews conducted during survey development noted that they have incorporated several preinjection and postinjection practices to reduce or alleviate discomfort or pain based on prior injection experiences, patient support group suggestions, and trial and error.

The noted disparity in LA SSA injection experiences calls for improvements in the consistency of the injection preparation and administration processes. Prior research has shown that nurses injecting octreotide intramuscularly have encountered challenges in reaching the intended site of injection, resulting in suboptimal control of CS symptoms following inadvertent subcutaneous injection [13]. A subsequent study to explore the pharmacokinetic profile of lanreotide administered subcutaneously versus intramuscularly revealed similar drug concentrations with both modes of administration, supporting the subcutaneous administration of lanreotide that is now standard clinical practice [14]. Moreover, novel formulations of SSAs, such as orally administered treatments, are imminent. An octreotide capsule for the treatment of acromegaly has shown positive safety and efficacy results and has been approved by the US Food and Drug Administration [15–17]. As administration practices for LA SSAs evolve and as additional administration modes may become available for patients with NETs, patients’ injection experience will be an important consideration when evaluating treatment success. Specifically, improving patients’ injection experiences may lead to higher patient satisfaction, confidence that the injection was administered correctly, and confidence that their medication is treating and controlling their disease.

Evaluations of patient priorities are increasingly important as patients are empowered to take more active roles in decision-making for their treatment pathways. In particular, patient preferences and treatment satisfaction have been identified as a critical component of patient autonomy and a foundation of shared decision-making between physicians and patients [18]. Treatment value frameworks also emphasize the importance of the patient voice and considering the outcomes that are important to patients when evaluating the value of new and emerging therapies, particularly because patient perspectives often differ from those of other stakeholders [19, 20]. Information gathered by the present study can be used for education of clinical and nursing staff and the broader medical community as the treatment landscape for NETs evolves to include several emerging therapies.

Some limitations inherent with all survey studies must be considered when these results are interpreted. First, the population surveyed may not be representative of the broader NET community. Interpretation of results should be made with caution and transparency of the study methodology, and the potential for self-selection bias, whereby particularly engaged patients may have participated in the study, must be considered. Specifically, the population was a convenience sample of patients recruited through a patient advocacy organization, and eligibility was established based on patient self-report; no corroboration with medical records or physicians was conducted. Invitations were distributed to members of CCF’s e-newsletter distribution list and access to the survey was dependent on screening questions to establish eligibility, patient report, and recollection of their most recent LA SSA injection. Patients with advanced disease may not be able to complete or be interested in online surveys, and their views may not be fully represented here. Although the qualitative phase of survey development did not include concept elicitation interviews, cognitive debriefing interviews provided an opportunity for patient feedback. Additional validation would be needed to assess the reliability and validity of the survey instrument. Finally, we conducted subgroup analyses to explore whether patients’ injection experiences were affected by therapy type; however, no significant differences were found and results may have been confounded by many other factors.

## Conclusions

In general, satisfaction with LA SSA injection is high among patients in this population. Patients’ specific experiences with LA SSA injections varied based on their perception of the administering nurses’ knowledge. As noted by patients in the cognitive interviews, and confirmed in serial surveys, more consistency or standard guidance in the administration of LA SSAs would be advisable. Informative dialogs between patients and administering nurses about expectations and processes would be beneficial to ensure proper administration and to avoid undesirable effects.

## Data Availability

The datasets generated during and analyzed during the current study are not publicly available but are available from the corresponding author on reasonable request.
